# News and Coming Events

**Published:** 1908-10-24

**Authors:** 


### News and Coming Events.

The Council of King's College, London, has appointed Dr. St. Clair Thomson as professor of laryngology in King's College Hospital Medical School.

Dr. H. G. Adamson, M.D.Lond., M.R.C.P.Lond., has been appointed physician-in-charge of the Skin Department at St. Bartholomew's Hospital.

Dr. A. R. Cushny will give a course of five lectures on Experimental Irregularities of the Heart, in the Physiological Lecture-room, University College, London, on Fridays, at 5 p.m., commencing on October 30.

At the conclusion of the business of the Convention of the American Hospital Association, Dr. Donald Mackintosh, M.V.O., of the Western Infirmary, Glasgow, was made an honorary member of the Association by a unanimous vote.

The annual dinner of the Royal Society of Medicine will be held at the Hôtel Cecil on Friday, December 4. It has been decided that on this occasion the price of the ticket to Fellows and Members of Sections shall be 7*s.* 6*d.*, exclusive of wine.

A sum of £20, collected by the Rev. W. J. Gomersall (in response to a letter published in the *Times*), has been presented to the Samaritan Fund of the Hospital for Sick Children, Great Ormond Street, in memory of the late Mrs. Ada S. Ballin.

A special meeting of the Governors of the Norfolk and Norwich Hospital was held at that institution on October 17, under the presidency of the Lord Lieutenant of Norfolk (Viscount Coke) to consider a report as to the steps necessary to maintain the efficiency of the hospital, and the question of the proposed amalgamation of the Norfolk and Norwich Eye Infirmary. The Board of Management was authorised to convene a public meeting to appeal for £45,000, a resolution approving of the scheme for the amalgamation being also adopted. This will involve an additional outlay of about £3,000, making the total estimated expenditure £48,000.

Mr. William Knight Treves, F.R.C.S.Eng., brother of Sir Frederick Treves, died at Cliftonville on October 14 at the age of sixty-five. For more than forty years he practised at Margate, and he had particularly identified himself with the study of tuberculosis. He was educated at St. Thomas's Hospital, and subsequently became a house-surgeon to that institution. He qualified M.R.C.S. in 1865, and F.R.C.S. in 1870. He had held the posts of surgeon, resident surgeon, and consulting surgeon to the Royal Sea Bathing Hospital at Margate.

Dr. Herbert Williams, the medical officer of health for the Port of London, states in his quarterly report to the Corporation that so far no case of actual or suspected cholera has arrived in the port. All vessels arriving from St. Petersburg are closely inspected, and all drinking water brought from that city is thrown overboard and the tanks thoroughly cleaned. Fifty-two alien immigrant vessels have arrived in the port, bringing 1,500 alien steerage passengers. The campaign against rats continues at the docks and on board ships, 540,280 having already been slaughtered. The names and addresses of all persons leaving the ships in London have been taken and communicated to the medical officers of the districts to which they were going. Persons remaining on the vessels have been visited daily, and inquiries have been made as to illness.

Mr. H. H. Clutton, F.R.C.S., has been elected one of the two representatives of the Royal College of Surgeons of England on the Senate of the University of London, in place of Mr. Henry T. Butlin, resigned. Dr. B. Moore, professor of bio-chemistry in the University of Liverpool, has been appointed an examiner in physiology for the second examination of the Conjoint Examining Board, and Mr. P. Sidney Spokes, M.R.C.S., has been re-elected a member of the Board of Examiners in dental surgery.

On October 1, at Guy's Hospital, an illuminated address on vellum was presented to Mr. Frederick Newland-Pedley, F.R.C.S., L.D.S., Consulting Dental Surgeon to the hospital, together with the sum of £205, subscribed for the purpose of establishing a Gold Medal for Practical Dentistry, as a permanent record of his valuable services to Guy's Hospital and Schools during a period of twenty-two years—namely, from 1885 to 1907. We learn from the *Guy's Hospital Gazette* that the first medal has been presented to Mr. Newland-Pedley, and that the award will be made annually to dental students.

At the opening of the winter session in the School of the Royal College of Surgeons in Ireland on October 15, Mr. J. Lentaigne, the President, laid stress on the fact that their school and college, which has been doing good work for more than a hundred years, is now the only undenominational teaching and diploma-giving body in Ireland, and, in their opinion, is also the only one which is not endowed or assisted in any way by the State. This is a glaring injustice. In face of the State-endowed competition of the other schools, the College School would have a hard struggle. They still hoped that Mr. Birrell would not ignore the claims of the only non-sectarian medical school in Ireland while largely endowing and adding to the endowment of bodies avowedly and ostentatiously sectarian.

The inaugural meeting of the winter session of the London School of Tropical Medicine was held on October 15 in the lecture-hall of the Royal Society of Medicine, under the chairmanship of Lord Crewe. Mr. P. J. Michelli, C.M.G., the secretary, presented the report of the Dean. This stated that the total number of students who had passed through the School since its opening in October 1899 was 849, showing an average of 94 per annum. About two-fifths of the students since the opening of the School either were in the Colonial medical services or had since joined them. Lord Crewe then delivered his address, in which he referred to the debt which the School owes to his predecessor Mr. Chamberlain, and to the responsibility which the Colonial Office owes both to the inhabitants of our tropical Colonies and Protectorates and to those officers and other servants whom it sends out to them.—Sir Clifford Allbutt thanked the tropical schools for the immensely stimulating influence they have had over the whole field of medicine.—Sir Patrick Manson said that nothing is so gratifying to the School as to have the support of the Government. The annual dinner of the School was held the same night at the Savoy Hotel under the chairmanship of Commander George Hodgkinson, R.N. Mr. Neville Chamberlain, in proposing "The School," said that in all his father's long administration at the Colonial Office, he did not think there was anything upon which he looked back with greater pride and satisfaction than his connection with the founding of that School.

Lieutenant-Colonel W. B. Leishman has been given an extension of his appointment as Professor of Pathology at the Royal Army Medical College.

Dr. E. H. Douty presided over the annual meeting of the Continental Anglo-American Medical Society, held in Paris on October 10, and at the annual dinner of the Society held that evening, Sir Dyce Duckworth took the chair.

The death is announced from Paris, at the age of fifty-seven, of M. Jean-Baptiste Pasteur, son of the great Pasteur. He served for some years in the Ministry for Foreign Affairs, and held various diplomatic appointments under the French Government.

The death occurred on October 14 of Dr. James Grey Glover, J.P., of Highbury, who was born at South Shields in 1832, graduated M.D. at Edinburgh in 1854, and was on the staff of the *Lancet* for forty years. He was especially noted for his advocacy in the columns of that journal of Lord Lister's antiseptic methods. He was one of the first three direct representatives of the profession on the General Medical Council, upon which he sat for sixteen years, and, in addition to conducting a large general practice, he acted as a J.P. for the County of London, and a visiting Justice of Holloway Prison. He was on the first Council of the Hospital Sunday Fund, and remained one of its keenest supporters. Dr. Glover also contributed to the ninth edition of the "Encyclopædia Britannica." His article on Homœopathy attracted a good deal of attention when that work was published on account of the uncompromising way in which the author attacked the bizarre principles and hypotheses which constitute that system.

The Royal Alexandra Infirmary, Paisley, has received a donation of £1,250 from Mrs. Stewart Clark, for the purpose of naming a bed in memory of her late husband, Mr. Clark, of Dundas Castle, who during his lifetime took an active part in the work of the institution and was one of its most generous supporters.

The Odontological Section of the Royal Society of Medicine gives notice that it is prepared to receive applications for grants in aid of the furtherance of scientific research in connection with dentistry. Particulars and forms of application may be obtained on applying to the Hon. Secretary Scientific Research Committee, Odontological Section, Royal Society of Medicine, 20 Hanover Square, London, W.

H.R.H. the Duchess of Albany, on October 19, formally opened the Infants' Health Exhibition, which has been organised by the Incorporated Institute of Hygiene at their premises, 34 Devonshire Street, Harley Street. Sir William Bennett, in asking the Duchess of Albany to open the Exhibition, said the Institute was inaugurated five years ago with the object of promoting the study and practice of personal and domestic hygiene, and in accordance with this principle it was now turning its attention to the diminution of infant mortality, which was mainly a matter of hygiene and proper feeding. The Duchess of Albany, after the opening ceremony, made a tour of inspection. The Exhibition, which will remain open until October 30, illustrates recent advances in connection with the health, comfort, and education of children. There is a model school nursery, and a model hospital ward from the Queen's Hospital for Children; and the City of London Lying-in Hospital is also represented by a model ward.

